# Genome-Wide Mapping Indicates That p73 and p63 Co-Occupy Target Sites and Have Similar DNA-Binding Profiles *In Vivo*

**DOI:** 10.1371/journal.pone.0011572

**Published:** 2010-07-14

**Authors:** Annie Yang, Zhou Zhu, Arminja Kettenbach, Philipp Kapranov, Frank McKeon, Thomas R. Gingeras, Kevin Struhl

**Affiliations:** 1 Department of Biological Chemistry and Molecular Pharmacology, Harvard Medical School, Boston, Massachusetts, United States of America; 2 Department of Genetics, Harvard Medical School, Boston, Massachusetts, United States of America; 3 Department of Cell Biology, Harvard Medical School, Boston, Massachusetts, United States of America; 4 Affymetrix, Santa Clara, California, United States of America; Institute of Genetics and Molecular and Cellular Biology, France

## Abstract

**Background:**

The p53 homologs, p63 and p73, share ∼85% amino acid identity in their DNA-binding domains, but they have distinct biological functions.

**Principal Findings:**

Using chromatin immunoprecipitation and high-resolution tiling arrays covering the human genome, we identify p73 DNA binding sites on a genome-wide level in ME180 human cervical carcinoma cells. Strikingly, the p73 binding profile is indistinguishable from the previously described binding profile for p63 in the same cells. Moreover, the p73∶p63 binding ratio is similar at all genomic loci tested, suggesting that there are few, if any, targets that are specific for one of these factors. As assayed by sequential chromatin immunoprecipitation, p63 and p73 co-occupy DNA target sites *in vivo*, suggesting that p63 and p73 bind primarily as heterotetrameric complexes in ME180 cells.

**Conclusions:**

The observation that p63 and p73 associate with the same genomic targets suggest that their distinct biological functions are due to cell-type specific expression and/or protein domains that involve functions other than DNA binding.

## Introduction

Eukaryotic organisms contain families of DNA-binding transcription factors comprised of structurally related proteins that are encoded by different genes. Individual members of the family are often co-expressed in the same cell, and in many cases they can associate with each other to generate heteromeric transcription factors. In addition, transcription factor families can exhibit cross-regulation, in which one family member affects the expression and/or function of another family member. In general, individual transcription factors within the family have both distinct and overlapping biological functions.

An important transcription factor family in mammalian cells includes the p53 tumor suppressor and two other proteins, p63 and p73, that are strikingly similarity to each other and less similar to p53 [1], [2]. p63 and p73 share ∼85% amino acid identity in their DNA binding domain, and they show strong structural and sequence similarity in their activation, oligomerization, and isoform-specific, C-terminal domains. p53 binds its target sites as a tetramer [3]–[6], and it is presumed that this is the case for p63 and p73. p63 and p73 exist as stable tetramers, and they interact efficiently to form heterotetramers [7], [8], although the DNA-binding activity of the heterotetramers has not been tested directly. Neither p63 nor p73 can form heterotetramers with p53, because p53 lacks a critical second helix in the tetramerization domain that is present in p63 and p73 [7], [8]. The various family members can co-exist in the same cell, and they exhibit cross-regulation [9]–[12]. In addition, p63 and p73 can transcriptionally regulate genes involved in DNA repair [13].

Despite the very high degree of similarity between p63 and p73, mouse knockout models reveal distinct and non-redundant physiological roles. p63-deficiency is associated with severe defects in epithelial development [14]–[16] and DNA damage responses in the female germline [17]. In contrast, p73 is implicated in various biological pathways including neurogenesis, inflammation, sensory pathways, and osteoblastic differentiation [18], [19] as well as genomic stability and tumor suppression [20]. The molecular basis for these distinct physiological roles is unknown.

There are multiple explanations, not mutually exclusive, for how two highly related members of the same protein family can have distinct biological functions. First, differences in tissue- and cell type-specific expression patterns can underlie distinct biological functions, even if the proteins are functionally equivalent. Differences in expression patterns might involve some or all of the structurally distinct isoforms that arise via alternative splicing, promoter usage, or 3′ end formation. Second, the two proteins can have distinct target specificities *in vivo*, either due to subtle differences in their DNA-binding domains and/or to differences in cooperative interactions with other DNA-binding proteins. Third, the two proteins can have functionally distinct domains that differentially mediate transcriptional activation or repression, interactions with co-activators or co-repressors, or interactions with other regulatory proteins. In cases where the proteins themselves are functionally distinct, the differences could be intrinsic to the protein sequence and/or reflect differences in phosphorylation or other post-translational modifications.

The *in vivo* binding behavior of highly related transcription factors in the same cells has rarely been examined in a global, unbiased manner. In the case of the ETS family of transcription factors, analysis of *in vivo* binding using genome-wide promoter microarrays revealed redundant and specific occupancy by individual members of the family [21]. Comparison of Stat5a and Stat5b, demonstrated that these highly homologous factors bind the same sites *in vivo*, albeit with different kinetics that may underlie differences in Stat5 biology [22]. A comparison of E2F family members in normal and tumor cells revealed very similar DNA-binding profiles in some cell types but not others [23].

In previous work, we used tiled microarrays covering the human genome to identify ∼5800 target sites for p63 in ME180, a cervical carcinoma cell line [24]. Here, we generate a DNA-binding profile of p73 in the same cells, thereby permitting a comparison of its *in vivo* target specificity to that of p63. We show that the p73 and p63 binding profiles are indistinguishable, with a similar p73∶p63 binding ratio at essentially all genomic loci. Furthermore, we show that p63 and p73 co-occupy DNA target sites *in vivo*, suggesting that p63 and p73 bind primarily as heteromeric complexes. The observation that p63 and p73 directly associate with the same set of genomic targets suggests that their distinct biological functions are due to cell-type specific expression and/or protein domains that involve functions other than intrinsic or cooperative DNA binding to target sites.

## Materials and Methods

### Chromatin immunoprecipitation (ChIP)

ME180 cells were grown in Dulbecco's Modified Eagle Medium (DMEM) supplemented with 10% Fetal Bovine Serum. Chromatin immunoprecipitation was performed with a mouse monoclonal antibody to p73 (11C12), essentially as described previously [24]. As assayed by Western blotting, this antibody interacts with p73, but shows no detectable cross-reactivity with recombinant p63 (Figure S1). Input and eluted material was treated with Pronase (1.5 µg/µl) for 2 hrs at 42°C and de-crosslinked by heating for 12 hours at 65°C. The samples were then purified using column purification (Qiagen PCR Purification kit) per manufacturer's instructions.

### Sequential CHIP

These were performed essentially as described in [25]. Briefly, chromatin from ∼3×10^8^ cells was immunoprecipitated with the 4A4 anti-p63 or 11C12 anti-p73 antibodies as described above. 10% of the eluted material was removed, de-crosslinked, and designated “1^st^ IP.” The remaining eluate was incubated with antibody-coupled protein A/G sepharose beads (11C12 for 4A4 1^st^ IP; 4A4 for 11C12 1^st^ IP), BSA (5 mg/ml), phage lambda DNA (25 µg/ml), and E. coli tRNA (50 µg/ml) in a total volume of 2 ml IP dilution buffer (approximately 10-fold dilution of eluate). Washes and elution were performed as described above, and eluted samples designated “2^nd^ IP.” Precleared chromatin from the 1^st^ IP was used as “input” DNA for both 1^st^ and 2^nd^ IP samples.

### Random primer amplification

Input and ChIP DNA was amplified by four rounds of primer extension (Round A) with random primers (GTTTCCCAGTCACGGTCNNNNNNNNN), using the following cycling conditions: 95°C, 4 min; 10°C, 5 min; +27°C at 1°C per 20 sec; 37°C, 8 min. Round A material was purified using column purification (Qiagen PCR Purification kit) and PCR amplified with primer B(GTTTCCCAGTCACGGTC). PCR program used was: 95°C, 3 min; followed by 30 cycles of 95°C, 30 sec; 40°C, 45 sec; 50°C, 45 sec, 72°C, 1 min; and a final extension at 72°C for 10 min. The samples were then purified using column purification (Qiagen PCR Purification kit) and ready for array hybridization protocols.

### Tiling array platform and generation of p73 binding sites

The high density, tiled whole genome arrays manufactured by Affymetrix covers essentially most of the non-repetitive DNA sequences of the human genome with (on average) one oligonucleotide pair every 35 bp. There are 7 chips in a full genome set and approximately 3,200,000 probe sets per chip (PM probes only). Array data from three biological replicates were scaled to target intensity of 500 and quantile normalized using Affymetrix Tiling Analysis Software (Version 1.1.02). A binding p-value was then determined for each genomic position by Wilcoxon rank sum test and binding sites were generated from those more significant than specified thresholds with a maximum gap of 500 and minimum run of 350. For every binding site, a binding enrichment score was computed from a smoothed “peak” estimator using the five genomic positions with the highest binding p-values (in the form of -10logP) within the region and one-step Tukey's biweight alogorithm. Data for p73 binding is stored at GEO (GSE18650).

### qPCR validation

qPCR was performed essentially as described previously [24], using an Applied Biosystems 7300 sequence detector for SYBR green fluorescence. The PCR program was: 95°C 10 min, followed by 40 cycles of 95°C, 30 sec, 60°C, 45 sec; 72°C, 1 min. Fold enrichment for a genomic region was determined relative to a non-enriched region (exon 3 of the histone H3 gene). The formula used was: fold enrichment = 1.9^−(ΔCTexpt-ΔCTref)^ where ΔCT is the cycle threshold (Ct) difference between ChIP DNA and input material, calculated for experimental and reference regions, and 1.9 is the mean primer slope. For each site, we calculated the occupancy units defined as the fold enrichment value minus background (H3 reference value set to 1). Based on our previous observations of p63 occupancy for various control negative regions [24], we defined validated targets as those regions showing greater than 2.5 occupancy units by qPCR as the negative controls were consistently below this cutoff. For “marginal” targets (i.e. 3 occupancy units or less), we required that at least 2 of the 3 replicates give greater than 2.5 occupancy units to avoid artificial inflation by a single replicate. This additional criterion was imposed because qPCR values for the p73 samples tended to be more variable than those for p63, likely due to the lower amounts of immunoprecipitated DNA.

### *De novo* motif discovery

For every binding site, we retrieved repeat-masked sequence and used *de novo* motif discovery algorithm MEME [26] to look for shared sequence motifs. MEME was run with the command line options “-mod oops -nmotifs 10 -evt 0.00001 –revcomp”. The background frequency was taken from the repeat-masked genome: A/T = 0.6 and C/G = 0.4.

### Sequence conservation analysis

Based on the multiz-8-way alignments for human, chimp, mouse, rat, dog, chicken, fugu and zebrafish [27], we generated overlaid versions of the human genome with corresponding sequences from the other seven species. In cases of more than one multiple alignment for a given human region (e.g., with different indels), we selected the one with the best alignment score. Percentage of sequence identity was calculated by counting the proportion of nucleotides in the p73-bound sequences with exact matches in the overlaid genome. Statistical significance was assessed with 1000 randomly sampled groups of the same number of sequences of the same length from the same chromosomes as p73 binding sites.

### Analysis of protein expression

Immunoblotting was performed with the 4A4 anti-p63 and 11C12 anti-p73 antibodies using standard procedures. Briefly, proteins were separated by SDS-PAGE, transferred to nitrocellulose membrane, blocked in 5% milk (in Tris-buffered saline with 0.05% Tween-20, TBST), and incubated with primary antibody followed by a horseradish peroxidase conjugated anti-mouse secondary (Jackson ImmunoResearch Laboratories). Chemiluminescent detection was performed with the SuperSignal Pico Chemiluminescent Substrate (Pierce) according to manufacturer's instructions.

## Results

### Mapping and verification of p73 binding sites

Chromatin from ME180 cells was immunoprecipitated with an anti-p73 monoclonal antibody (11C12) that shows no detectable cross-reactivity with p63 (Figure S1). The immunoprecipated DNA was hybridized to the Affymetrix Human Tiling 2.0R array set, interrogating the non-repetitive sequences of the entire human genome. Data from three biological replicates were combined, and we identified 488 p73 sites at a significance threshold of P≤10^−5^, the same cut-off used for the p63 analysis previously described [24]. The number of p73 binding sites is considerably fewer than the 5800 p63 sites identified in the same manner, but this may reflect the relative expression levels of the two proteins. In this regard, p63 is more abundant than p73 in squamous epithelial cells [28].

We used “real-time,” quantitative polymerase chain reaction (qPCR) to verify p73 enrichment at several targets identified from the ChIP-Chip experiment. Five of five sites with *P*≤10^−5^ were verified as “true positives,” defined as showing at least an average 2.5-fold enrichment in three biological replicates, relative to a negative control region. We also tested regions with lower binding scores and verified four of five sites for 10^−5^≤*P*≤10^−4^, and eight of nine targets for 10^−4^≤*P*≤10^−3^ (Table S1). These results indicate many p73 sites from the lower stringency cut-offs represent true p73 binding targets, consistent with the notion that transcription factor binding affinities *in vivo* represent a continuum, rather than simple presence or absence of binding [29], [30]. Nevertheless, to facilitate comparisons with p63 data, we chose the P≤10^−5^ cutoff for most of our subsequent analyses.

**Figure 1:**
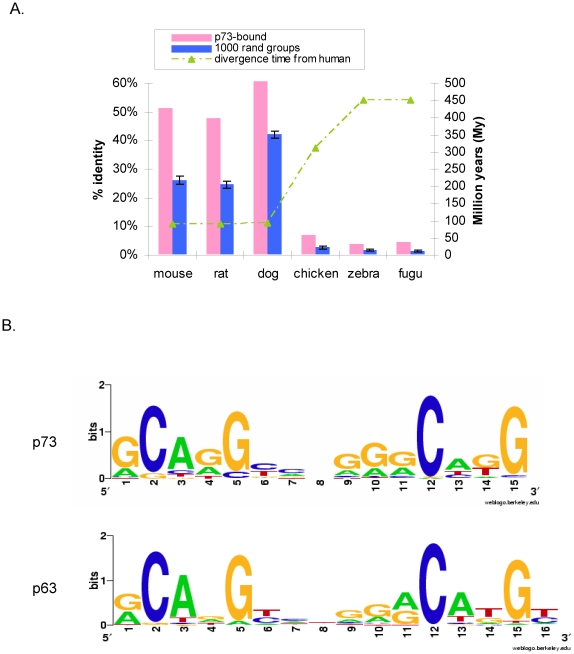
Evolutionary conservation and DNA sequence motif of p73-bound sites in ME180 cells. (A) Evolutionary conservation is defined as the total percent identities of p73-bound sequences and 1000 groups of randomly selected comparable genomic sequences across multiple species. Error bars correspond to standard deviation from 1000 randomly sampled groups. (B) The DNA sequence motif for p73 derived *de novo* from the genomic binding sites is compared to the motif for p63 [24] and p53. The p73 motif is essentially identical to the p63 response (CompareACE score = 0.95) [34].

Many p73 binding sites in ME180 are located in the vicinity of annotated, full-length transcripts, and they exhibit a preference for the 5′-ends of genes, with 8.4% and 22.4% of the 488 sites (P≤10^−5^) located within 1 kb and 5 kb upstream of the transcriptional start, respectively. p73 binding sites included previously reported p73 targets, such as the PUMA, mdm2, and p63 genes, and they show strong evolutionary conservation (Fig. 1A). The consensus motif derived from the identified p73 target sites is very similar to the motif for p53 and indistinguishable from the p63 response element in ME180 cells (Fig. 1B)[24]. However, this motif occurs numerous times in the mammalian genome, and it is a poor predictor of where the proteins actually bind *in vivo* and hence whether p63 and p73 have similar genomic DNA-binding profiles [24].

### p63 and p73 have indistinguishable DNA binding profiles in ME180 cells

**Figure 2:**
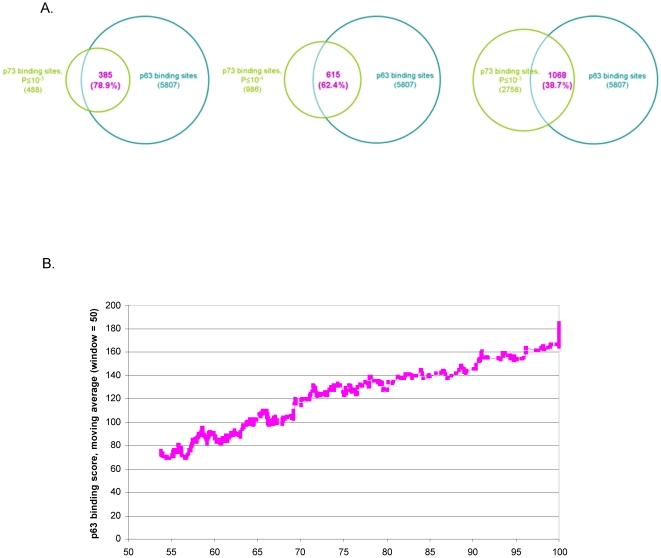
p63 and p73 have very similar DNA-binding profiles in ME180 cells. (A) Overlap of p63 and p73 binding sites at three different significance thresholds. p63 binding sites were identified in our previous work [24], and percentages of overlap are expressed with respect to p73 sites. (B) Correlation between p63 and p73 binding enrichment scores, which were defined as described in Methods. p63 scores are plotted as a moving average (window size = 50).

Comparison of the p73 binding profile with the previously described p63 binding profile [24] reveals a striking overlap between p63 and p73 binding sites in ME180 cells. Nearly 80% of p73 targets at a significance threshold of P≤10^−5^ overlap with p63 binding sites identified in our previous work (Table S2). The percentage overlap is above 60% even at the lower stringency cut-off of P≤10^−4^ (for p73), supporting the similarity between p63 and p73 binding, and a further indication that sites in this range are bona fide p73 targets. A comparison of p63 and p73 binding enrichment scores shows a strong correlation (Pearson correlation = 0.414), with p63 generally showing higher scores than p73 (Fig. 2). These observations are reminiscent of p63 binding in the presence (+) or absence of (−) actinomycin D, where drug treatment reduces p63 protein levels and association with DNA, but does not alter binding specificity [24].

**Figure 3:**
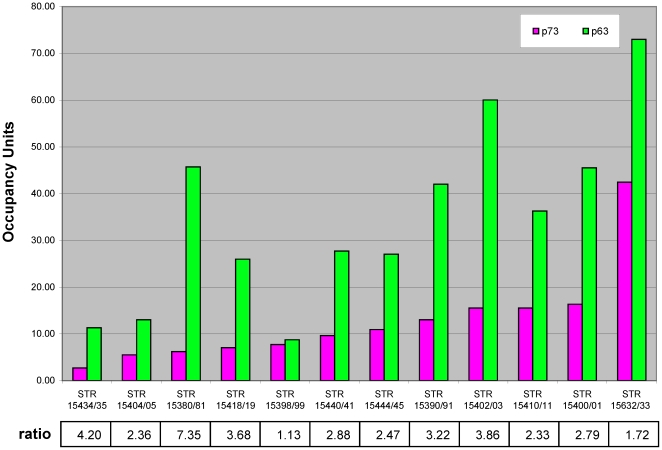
Comparable ratio of p63 and p73 association with target sites. Shown is the average occupancy value from 3 biological replicates. The ratio of p63 to p73 occupancy at each target site is indicated.

Although p63 and p73 have similar DNA binding profiles, the above analysis does not address the possibility that a subset of sites are differentially bound by one of the proteins. To examine this possibility, we used quantitative PCR analysis to determine the relative occupancy of p63 and p73 at selected p73 targets that had a range of array-based p63 binding scores (Fig. 3). All 12 sites that were validated for p73 enrichment show clear p63 binding, including sites with p63 binding scores below the 10^−5^ cutoff used previously to define p63 targets [24]. In this regard, the p73 binding data is useful for identifying true p63 target sites that were false negatives at the cutoff chosen in the previous analysis. We also examined putative p73 target sites (i.e. passed the 10^−5^ cutoff) that had very low levels of p63 occupancy (*P*<10^−2^). In all such cases tested, these putative p73-only sites showed no detectable levels of either p63 or p73, indicating that these were false positives from the p73 array results. Most importantly, for all sites tested, the relative occupancy of p63 and p73 appeared similar, with p63 enrichment being approximately 2–4 fold higher than that of p73 (Fig. 3). Thus, we could not demonstrate evidence of unique binding sites for p73 in ME180 cells.

### p63 and p73 co-occupancy *in vivo*

The observation that p73 and p63 have indistinguishable binding profiles in ME180 cells does not indicate whether these two factors are simultaneously bound at the same loci. We therefore used sequential chromatin immunoprecipitation to determine co-occupancy of p63 and p73 *in vivo* [25]. Specifically, we first performed an immunoprecipitation with antibodies for one factor (i.e. p63 or p73), eluted the protein-DNA complexes, and then immunoprecipitated the resulting sample with antibodies for the other factor (i.e. p73 or p63, respectively). If two factors co-occupy a DNA site, binding enrichment after the sequential immunoprecipitations should be higher than enrichment observed in the single immunoprecipitation [25].

**Figure 4:**
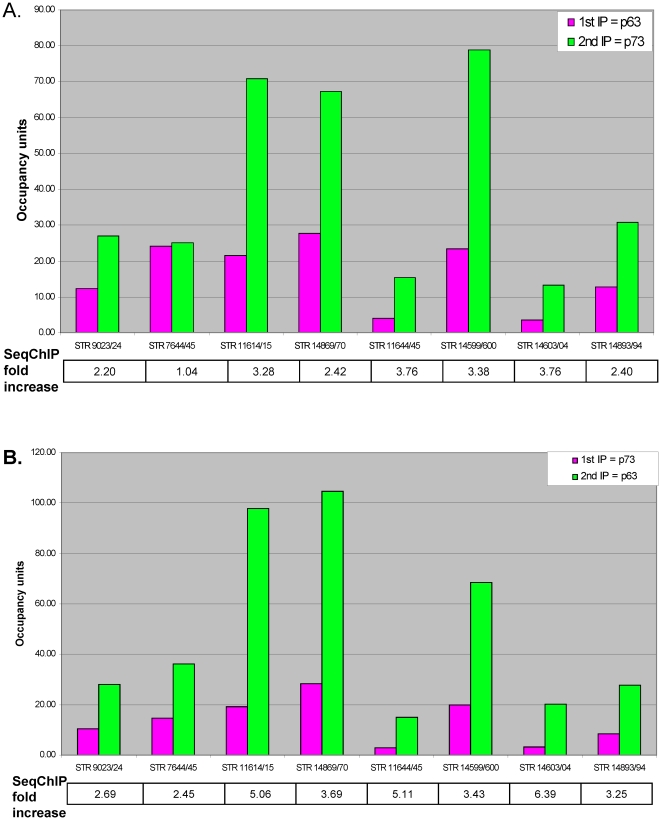
p63 and p73 co-occupancy *in vivo*. Sequential chromatin immunoprecipitation (SeqChIP) samples were analyzed by quantitative PCR (qPCR) for p63 and p73 enrichment at various targets. Shown is the average occupancy value from 3 biological replicates. (A) p63 immunoprecipitation (1^st^ IP) followed by p73 immunoprecipitation (2^nd^ IP). (B) p73 immunoprecipitation (1^st^ IP) followed by p63 immunoprecipitation (2^nd^ IP). The fold-increase (2^nd^ IP over 1^st^ IP) in enrichment after sequential ChIP is indicated.

For all eight targets tested, co-occupancy of p63 and 73 was observed when the first immunoprecipitation was performed with p73 (Fig. 4A). Specifically, the fold-enrichments in the sequential immunoprecipitations were 2.5–6 fold higher than in the p73 immunoprecipitation. In the reciprocal experiment in which p63-bound targets were immunoprecipitated first, seven of eight targets showed increased enrichment upon subsequent immunoprecipitation with p73 (Fig. 4B). In all cases, the increase in fold-enrichment upon sequential immunoprecipitation was higher when p73-bound targets were purified first. Indeed, in the one instance where we could not demonstrate an increase in fold enrichment upon sequential immunoprecipitation, this occurred only when p63 is the first factor immunoprecipitated. This asymmetry in sequential ChIP results indicates that p63 and p73 partially co-occupy their target sites *in vivo* [25]. Partial co-occupancy is likely to be due to the fact that p63 protein levels markedly exceed those of p73 (see Discussion).

**Figure 5:**
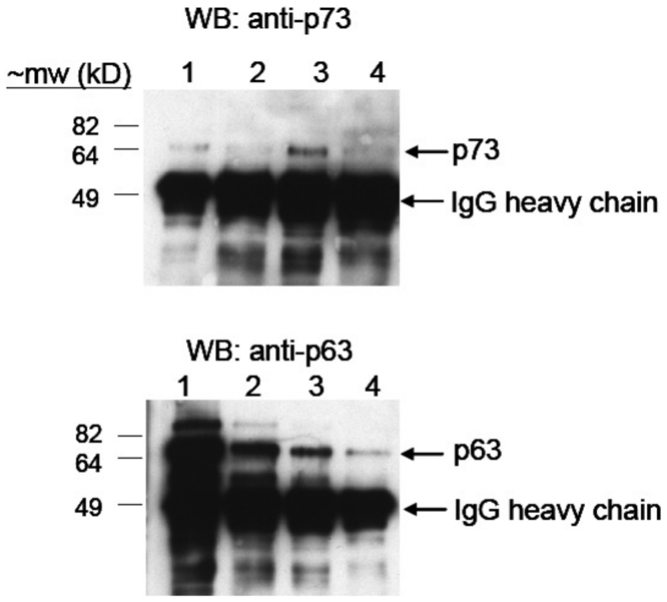
Western blot (WB) analysis of sequential chromatin immunoprecipiations for p63 and p73. Eluates were probed with anti-p73 (top gel) or anti-p63 (bottom gel) antibodies. lane 1: anti-p63 1st IP; lane 2: anti-p73 2nd IP; lane 3: anti-p73 1st IP; lane 4: anti-p63 2nd IP. Mw, molecular weight; kD, kiloDaltons, IgG, immunoglobulin.

p63 and p73 co-immunoprecipitate in cell-free extracts [28], presumably because they can form heterotetramers [7], [8]. Consistent with this observation, co-immunoprecipitation of p63 and p73 was observed in samples from the first and second immunoprecipitations used in the sequential ChIP analysis above (Fig. 5). Thus, it is likely that p63 and p73 have indistinguishable genomic targets in ME180 cells primarily due to the ability of these proteins to form DNA-binding heterotetramers.

## Discussion

### p73 and p63 have indistinguishable genomic targets and bind as a heteromeric complex in ME180 cells

Several observations indicate that p63 and p73 have indistinguishable genomic targets in ME180 cells. First, there is a striking overlap between p63 and p73 targets (Tables S1 and S2), and a strong correlation between p63 and 73 binding scores based on the genome-wide array data (Fig. 2). Second, among the small subset of target sites that appear from the genome-wide array experiments to be differentially bound by p63 and p73, we examined the best candidates for targets that are bound by p73, but not p63, and found that all tested were either false positives (for p73) or false negatives (for p63). Thus, most (and perhaps all) sites that appear to be differentially bound by p73 and p63 are explained by being false positives or negatives in one of the analyses. Third, for all loci tested by quantitative PCR, the relative binding ratio of p63 and p73 is similar. By definition, differential binding by p63 and p73 to a given target site should result in a skewed binding ratio when compared to typical target sites. Thus, our analyses indicate that there are few, if any, target regions that are differentially bound by p63 and 73, and that any differences in relative binding by these two proteins among target sites are subtle.

The sequential ChIP experiment demonstrates that p73 and p63 co-occupy all sites tested, and hence most (and perhaps all) genomic targets. Such co-occupancy indicates that p63 and p73 can bind to their targets as heteromeric complexes. As p63 and p73 form stable heterotetramers in solution [7], [8], the observed co-occupancy strongly suggests that these proteins can bind DNA as heterotetramers. Formally, we cannot exclude the possibility that co-occupancy arises from independent binding of p63 and p73 to distinct sites in close proximity, although this situation is likely to be rare, especially given that few target sites have multiple copies of the DNA-binding motif.

The ability of p63 and p73 to bind as heterotetramers provides a simple explanation for why they have indistinguishable binding specificites in ME180 cells. However, our experiments do not indicate that the similar target profiles of p63 and p73 are due exclusively to binding by heterotetrameric complexes. It is possible that p63 and p73 homotetramers have similar target specificity to each other and likely (although not necessarily) to the heterotetrameric complexes.

Numerous genome-wide ChIP experiments reveal that fold-enrichments for association of a given protein to target sites vary over a wide range. This indicates that most target sites are not fully occupied by the transcription factor, and hence that the concentration of the protein is limiting for binding, except perhaps for the strongest sites. As a consequence, and as observed in our previous work on p63 [24], intracellular protein concentration affects fold-enrichments and hence the number of target sites identified via thresholding, although it does not affect site specificity. Thus, higher levels of p63 vs. p73 is likely to explain why the number of identified p73 target sites appears to be far lower than the number of p63 sites, even though the two proteins have indistinguishable target specificity.

As p63 and p73 can bind as a heteromeric complex, the difference in protein levels affects the stoichiometry of the complexes. As these proteins, like p53, bind as tetramers, p73 will be typically under-represented in hetero-tetramers, and there should be a significant number of p63 homo-tetramers in ME180 cells. In accord with theoretical considerations of sequential ChIP experiments [25], we observe that the co-occupancy of p73 and p63 is partial and that higher fold-enrichments occur when p73 is immunoprecipitated first.

### Biological implications

Mutational analysis in mice indicates that p63 and p73 control very different physiological processes [1], [14]–[16], [19]. Our results suggest that, in cell types expressing both proteins, p63 and p73 will directly affect the same set of target genes. At such genes, the target sites will be bound by essentially the same ratio of heterotetrameric and homotetrameric complexes, with the ratio being determined by the relative concentrations of p63 and p73 and the DNA-binding activity of the different types of complexes. The ratio of the various complexes at target sites can be modified by physiological conditions that affect one or both proteins. In such cell types, if p73 and p63 have differential functions, these are unlikely to be due to the selection of target genes, but rather differences in transcriptional functions (i.e. activation, repression, or interactions with co-regulatory factors) of the two proteins.

Other cell types express either p73 or p63, but not both. In such cell types, phenotypic effects can only be observed when the gene encoding the expressed protein is mutated. Similarly, if one protein is much more prevalent than the other, as is the case in ME180 cells, it is likely that mutation of the more abundant protein will cause stronger phenotypic effects. Thus, many, and perhaps most, of the distinct biological functions of p63 and p73 are likely to reflect differences in cell-type-specific expression.

The DNA-binding profiles of p73 and p63 in cell types that express only one of these proteins are unknown. In such cell types, it is likely, that p63 and p73 will associate with some sites in common, but interact differentially with other genomic regions. However, individual DNA-binding proteins typically have cell-type specific binding profiles, because binding to chromatin templates in mammalian cells generally requires cooperative interactions with other proteins [24]. Such cooperativity could involve direct protein-protein contact, common interactions with a third factor, or synergistic recruitment of chromatin-modifying factors. Thus, it is unclear whether, on their own, p73 and p63 recognize the same set of target genes. Indeed, it remains possible that p73 and p63 are functionally equivalent proteins, whose distinct biological functions reflect differences in expression.

Our findings address reports of p63 and p73 antagonism in head and neck squamous cell carcinomas [28], [31], [32], which are a similar cell type to ME180 cervical carcinomas. Specifically, ΔNp63 suppresses TAp73 activation of apoptosis target genes, Puma and Noxa, and it was proposed that p63, which is overexpressed in these cells, directly competes for binding to these promoters and blocks p73 occupancy [28]. Our results invalidate the notion that p63 and p73 DNA binding is mutually exclusive. Instead, they suggest that this reflects competition between p63 homotetramers, p73 heterotetramers, or p63/p73 heterotetramers that have different transcriptional activities. Lastly, our results are relevant to numerous studies reporting imbalances of p63 and p73 isoforms in cancer [33]. In particular, apparent roles of p63 and p73 will be strongly influenced by the relative levels of these two family members, as well as the specific isoforms of each protein.

## Supporting Information

Table S1 (1.02 MB PDF)

Table S2 (0.06 MB PDF)

Figure S1 Figure S1
(0.27 MB PDF)
