# Supplementary material for: Genome-Wide Mapping Indicates That p73 and p63 Co-Occupy Target Sites and Have Similar DNA-Binding Profiles In Vivo
Source: PLoS One. 2010 Jul 14;5(7):e11572. doi: 10.1371/journal.pone.0011572 (PMC2904373; doi:10.1371/journal.pone.0011572)
Supplement: Table S1 — (1.02 MB PDF) [file pone.0011572.s001.pdf]

# Supplementary Materials

**Table 1.** qPCR verification of p73 binding sites in ME180 cells

| Threshold | Target ID | binding score | p73_Avg | p73_B1 | p73_B2 | p73_B3 | verified? |
|---|---|---|---|---|---|---|---|
| $p\text{-val} \leq 10^{-3}$ | STR 15380/81 | 29.99 | 6.23 | 4.59 | 6.77 | 7.33 | Y |
| | STR 15382/83 | 31.93 | 2.81 | 5.30 | -0.10 | 3.24 | Y |
| | STR 15384/85 | 32.02 | 2.10 | 6.43 | -0.14 | 0.00 | N |
| | STR 15388/89 | 32.57 | 17.59 | 19.64 | 3.64 | 29.50 | Y |
| | STR 15390/91 | 33.00 | 13.04 | 10.86 | 9.80 | 18.46 | Y |
| | STR 15398/99 | 35.34 | 7.73 | 1.94 | 4.87 | 16.38 | Y |
| | STR 15400/01 | 35.66 | 16.33 | 16.36 | 11.22 | 21.42 | Y |
| | STR 15402/03 | 37.13 | 15.55 | 21.68 | 13.57 | 11.39 | Y |
| | STR 15404/05 | 37.62 | 5.52 | 4.79 | 1.57 | 10.21 | Y |
| $p\text{-val} \leq 10^{-4}$ | STR 15410/11 | 40.82 | 15.56 | 19.21 | 12.23 | 15.25 | Y |
| | STR 15416/17 | 42.57 | 1.36 | -0.01 | 1.07 | 3.02 | N |
| | STR 15418/19 | 43.44 | 7.07 | 8.65 | 5.98 | 6.58 | Y |
| | STR 15632/33 | 46.45 | 42.47 | 77.45 | 26.07 | 23.90 | Y |
| | STR 15422/23 | 44.47 | 15.05 | 13.66 | 11.82 | 19.67 | Y |
| $p\text{-val} \leq 10^{-5}$ | STR 15428/29 | 52.41 | 6.42 | 6.30 | 2.36 | 10.61 | Y |
| | STR 15432/33 | 57.75 | 17.07 | 13.66 | 6.72 | 30.84 | Y |
| | STR 15434/35 | 61.59 | 2.70 | 1.09 | 3.17 | 3.83 | Y |
| | STR 15440/41 | 72.01 | 9.65 | 3.21 | 6.77 | 18.97 | Y |
| | STR 15444/45 | 88.63 | 10.95 | 3.47 | 9.99 | 19.38 | Y |

[a] binding enrichment scores are indicated for each biological replicate (B1,B2, B3) as well as the average (avg) fold enrichment of all three replicates

[b] see Methods for details on binding enrichment score

[c] a target is verified (Y = Yes; N=No) if avg fold enrichment is >2.5 fold AND 2 independent replicates each show >2.5 fold
