# Supplementary material for: Genome-Wide Mapping Indicates That p73 and p63 Co-Occupy Target Sites and Have Similar DNA-Binding Profiles In Vivo
Source: PLoS One. 2010 Jul 14;5(7):e11572. doi: 10.1371/journal.pone.0011572 (PMC2904373; doi:10.1371/journal.pone.0011572)
Supplement: Table S2 — (0.06 MB PDF) [file pone.0011572.s002.pdf]

Supplementary Table 2. The distribution of p73 binding sites in ME180 cells relative to p63 and well-characterized genes [a, b]

**TFBS**[c]	$P \le 10^{-3}$ [d]	$P \le 10^{-4}$ [d]	$P \le 10^{-5}$ [d]	Max (rand)[e]	Min (rand)[f]
Total	2758	986	488	N/A	N/A
overlap with p63	1068 (38.7%)	615 (62.4%)	385 (78.9%)	N/A	N/A
gene vicinity	1905 (69.1%)	637 (64.6%)	308 (63.1%)	42.6%	38.2%
up5K	612 (32.1%)	173 (27.2%)	69 (22.4%)	13.7%	9.3%
up1K	406 (21.3%)	88 (13.8%)	26 (8.4%)	3.6%	1.7%
all introns	1091 (57.3%)	426 (66.9%)	230 (74.7%)	90.1%	86.3%
intron 1	613 (32.2%)	220 (34.5%)	107 (34.7%)	30.1%	24.3%
intron 2	197 (10.3%)	80 (12.6%)	45 (14.6%)	21.0%	15.9%
intron 3	129 (6.8%)	57 (8.9%)	33 (10.7%)	15.8%	11.3%
all exons	542 (28.5%)	133 (20.9%)	45 (14.6%)	7.5%	4.0%
exon 1	365 (19.2%)	72 (11.3%)	15 (4.9%)	2.1%	0.7%
exon 2	79 (4.1%)	23 (3.6%)	11 (3.6%)	1.4%	0.3%
exon 3	29 (1.5%)	8 (1.3%)	5 (1.6%)	1.2%	0.1%
5' UTR	171 (9.0%)	34 (5.3%)	5 (1.6%)	1.4%	0.2%
[illegible]	36 [illegible]	10 [illegible]	8 [illegible]	3.3%	1.4%

[a] Gene structure information was taken from UCSC knownGene and RefSeq annotations.

[a] Gene structure information was taken from UCSC knownGene and RefSeq annotations.

[b] distance calculations and gene associations are relative to the midpoint of p73 binding sites

[c] TFBS = transcription factor binding site

[d] P-value threshold used in binding site generation.

[e] maximum percentage from 1000 random runs; data from Yang et al, 2006 (ref 24);N/A = not applicable

[f] minimum percentage from 1000 random runs; data from Yang et al, 2006 (ref 24);N/A = not applicable
