# Supplementary material for: Genome-Wide Mapping Indicates That p73 and p63 Co-Occupy Target Sites and Have Similar DNA-Binding Profiles In Vivo
Source: PLoS One. 2010 Jul 14;5(7):e11572. doi: 10.1371/journal.pone.0011572 (PMC2904373; doi:10.1371/journal.pone.0011572)
Supplement: Figure S1 — (0.27 MB PDF) [file pone.0011572.s003.pdf]

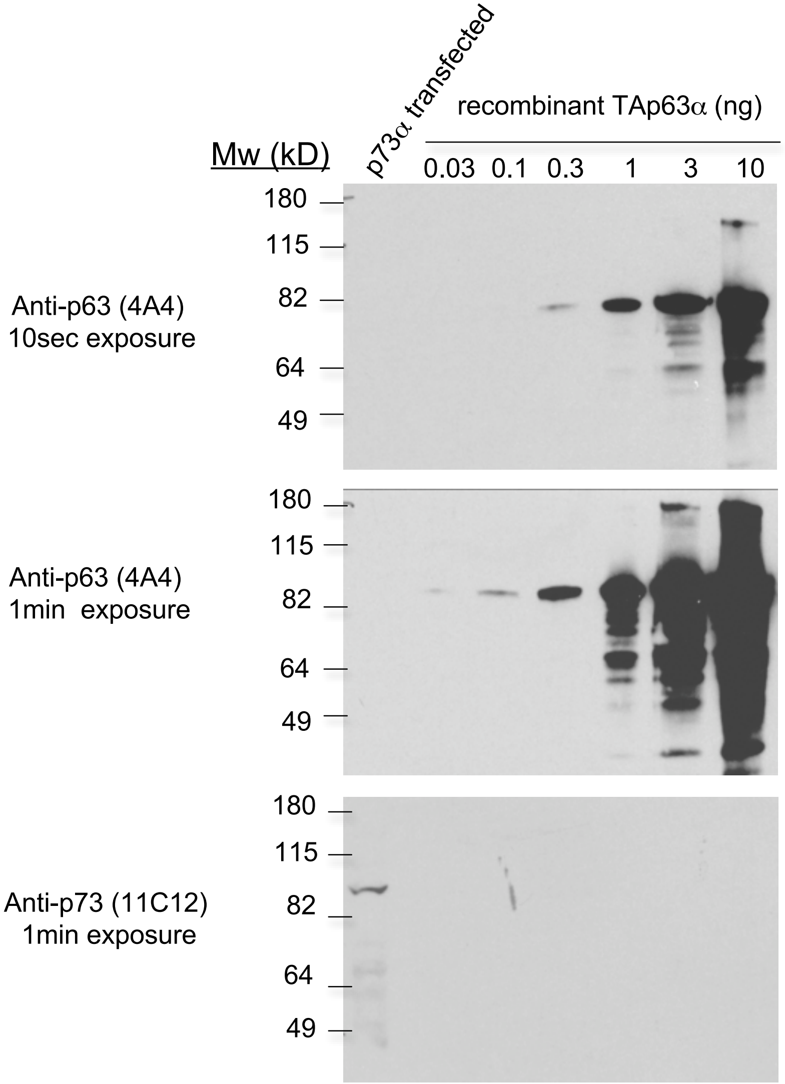

Supp Figure 1. Specificity of p63 and p73 monolconal antibodies. Immunoblots of recombinant TAp63α protein or HeLa cells transfected with a p73α cDNA were probed with monoclonal antibodies for p63 and p73, as indicated.
